# Prognostic Factors and Survival Outcomes in Parotid Gland Mucoepidermoid Carcinoma: A Systematic Review with Meta-Analysis and Workflow Proposal

**DOI:** 10.3390/cancers18071146

**Published:** 2026-04-02

**Authors:** Giovanni Salzano, Veronica Scocca, Luigi Angelo Vaira, Jerome R. Lechien, Alfonso Scarpa, Stefania Troise, Giovanni Dell’Aversana Orabona

**Affiliations:** 1 Maxillofacial Surgery Unit, Department of Neurosciences, Reproductive and Odontostomatological Sciences, University Federico II of Naples, Via Pansini 5, 80131 Naples, Italy; giovanni.salzano2@unina.it (G.S.); stefania.troise@unina.it (S.T.); giovanni.dellaversanaorabona@unina.it (G.D.O.); 2 Maxillofacial Surgery Operative Unit, Department of Medical, Surgical and Experimental Sciences, University of Sassari, 07100 Sassari, Italy; lavaira@uniss.it; 3 Department of Surgery, University of Mons, 7301 Mons, Belgium; jerome.lechien@umons.ac.be; 4 Department of Medicine, Surgery and Dentistry, University of Salerno, 84081 Salerno, Italy; ascarpa@unisa.it

**Keywords:** salivary gland, parotid, mucoepidermoid carcinoma, salivary gland neoplasms, survival, systematic review

## Abstract

**Objective:** Mucoepidermoid carcinoma (MEC) is the most common malignant tumour of the parotid gland. This systematic review and meta-analysis aims to evaluate treatment strategies, survival, recurrence, and prognostic factors in primary parotid MEC. **Materials and Methods:** A systematic review was conducted following PRISMA guidelines. PubMed/MEDLINE, the Cochrane Library, Scopus, and Google Scholar were searched to identify eligible observational studies and clinical trials on primary parotid MEC. Pooled estimates of overall survival (OS), disease-specific survival (DSS), and local, regional, and distant recurrence rates were calculated. Prognostic factors associated with survival and recurrence were analysed. **Results:** Twenty-one studies involving 7192 patients were analysed. Histologic grade was low in 32.2%, intermediate in 41.8%, and high in 26.1%. Surgical treatment included total parotidectomy (2606 patients) and superficial parotidectomy (1642), with facial nerve preservation achieved in 1993 of 4111 reported cases. Positive margins occurred in 18% of patients, and postoperative radiotherapy was administered in 50%. Mean follow-up was 72.6 months. Pooled OS rates were 100% at 1 year, 90% at 5 years, and 70% at 10 years; DSS was 100% at 5 years and 90% at 10 years. Recurrence rates were 10% local, 0% regional, and 10% distant. High-grade histology, advanced T/N stage, positive surgical margins, and intraparotid lymph node metastasis were associated with poorer outcomes. **Conclusions:** Parotid MEC generally has favourable short- and intermediate-term outcomes. Based on evidence and institutional experience, we propose a grade-based workflow integrating tumour grade, T/N status, and adverse pathological features to guide surgical extent, elective neck dissection, and adjuvant radiotherapy. Prospective studies with standardized reporting are needed.

## Simple Summary

Mucoepidermoid carcinoma (MEC) is the most common malignant tumor of the parotid gland, but because of its overall rarity, evidence regarding prognosis and optimal treatment remains limited and fragmented. This systematic review and meta-analysis aimed to summarize the available literature on primary parotid MEC, focusing on survival outcomes, recurrence patterns, treatment strategies, and prognostic factors. Twenty-one retrospective studies involving more than 7000 patients were included. Overall, parotid MEC showed favorable short- and intermediate-term survival, although outcomes were significantly worse in patients with high-grade tumors, advanced T or N stage, positive surgical margins, and intraparotid lymph node metastasis. These findings highlight the importance of histologic grade and pathologic risk factors in treatment planning and long-term follow-up. More standardized prospective studies are needed to improve risk stratification and guide management.

## 1. Introduction

Mucoepidermoid carcinoma (MEC) represents the most common malignant tumour of the salivary glands, accounting for approximately 30–40% of all salivary gland malignancies and occurring in the parotid gland in nearly 70% of cases. Several environmental and lifestyle-related risk factors have been described, including tobacco use, exposure to nickel and lead, steroid hormones, ionizing radiation, obesity, and dietary habits [1,2].

Management of MEC varies considerably depending on tumour size, grade, anatomical location, stage, and the presence of regional or distant metastases. Therapeutic strategies range from wide local excision alone to wide excision combined with neck dissection and/or adjuvant radiotherapy.

Low-grade MEC is generally associated with indolent biological behavior [3] and is commonly managed with complete surgical excision, without a routine need for elective neck dissection (END) or adjuvant radiotherapy, unless margins are close or positive. Observation of the cervical lymphatic basins is typically sufficient in clinically node-negative (cN0) patients.

By contrast, management of intermediate-grade MEC remains controversial, with no clear consensus regarding the indications for END or adjuvant treatments. Neck dissection may be performed when regional metastasis is clinically evident, in high T-stage disease, or when the tumour displays mixed or ambiguous histopathological characteristics. Biologically, intermediate-grade MEC behaves between low- and high-grade tumours [4]. In contrast, high-grade MEC demonstrates more aggressive biological behaviour and frequently requires multimodal treatment, including wide local excision, elective or therapeutic neck dissection, and postoperative radiotherapy to reduce the risk of local recurrence. In patients with clinically or radiologically evident nodal metastases (cN+), neck dissection is always indicated. Moreover, even in cN0 patients, END is often recommended due to the 30–50% risk of occult lymph node metastasis in high-grade tumours [5,6].

Despite MEC being the most common malignant neoplasm of the parotid gland, its overall rarity, diagnostic variability, and challenges in grading hinder the ability to evaluate outcomes consistently across studies.

The aim of this systematic review and meta-analysis is to evaluate prognostic factors, treatment strategies, and oncologic outcomes in patients with parotid MEC, with a particular focus on identifying predictors of survival and recurrence.

## 2. Materials and Methods

This systematic review and meta-analysis was performed in compliance with the Preferred Reporting Items for Systematic Reviews and Meta-Analyses (PRISMA) statement. As the study was based exclusively on previously published data, institutional review board approval and informed consent were not required. The review protocol was prospectively registered in the PROSPERO database (registration ID: 1244504).

### 2.1. Search Strategy

A comprehensive literature search was carried out to identify studies published between 1950 and 2025. The following electronic databases were searched: PubMed/MEDLINE, Cochrane Library, Scopus, Embase, and Google Scholar. Two reviewers (V.S. and G.S.) independently performed the search. Search terms, keywords, and MeSH headings were adapted to the indexing system of each database. The main search strategy combined terms related to mucoepidermoid carcinoma and the parotid gland. In addition, the reference lists of the selected articles were manually screened to identify any further relevant studies through backward citation searching. The electronic search was conducted between 1 November 2025 and 11 November 2025.

### 2.2. Eligibility Criteria

Eligibility was defined according to the PICOS framework. Studies including mixed populations, such as patients with MEC arising from other major salivary glands, were considered eligible only when data specific to primary parotid gland MEC could be clearly extracted.

### 2.3. Inclusion Criteria

#### 2.3.1. Patients (P)

Patients with primary parotid gland mucoepidermoid carcinoma.

#### 2.3.2. Intervention (I)

Any surgical or non-surgical intervention, including but not limited to parotidectomy, neck dissection, radiotherapy, chemoradiotherapy, and targeted therapies.

#### 2.3.3. Comparison (C)

None.

#### 2.3.4. Outcomes (O)

Primary outcomes:

- 1y, 5y, 10y-Overall survival (OS).
- 1y, 5y, 10y-Disease-free survival (DFS).
- Local, regional, or distant recurrence rates.

Secondary outcomes:

- Pathological and clinical prognostic factors (e.g., grade, T stage, nodal status, margin status, PNI, LVI).
- Treatment strategies.
- Tumour characteristics at diagnosis, including histologic grade and T/N stage.

#### 2.3.5. Study Design (S)

Retrospective and prospective cohort studies, case–control and cross-sectional studies and RCTs.

### 2.4. Exclusion Criteria

Studies were excluded if the full text was unavailable, if they enrolled fewer than five patients, or if they were published in languages other than English. Review articles, case reports, conference abstracts, letters, and book chapters were also excluded. In addition, studies focusing exclusively on recurrent disease, as well as mixed cohorts without separately extractable data for parotid MEC, were not considered eligible.

### 2.5. Data Collection Process

All references retrieved from the database search were imported into EndNote^®^ 21 (version 21.5), where duplicates were identified and removed. Screening was then performed in two stages, first by title and abstract and subsequently by full-text review of potentially eligible studies. Disagreements between the two reviewers were resolved through discussion until consensus was reached.

Potential duplicate cohorts were identified by comparing study periods, institutions, and patient characteristics; in cases of overlap, the most comprehensive dataset was included.

Data extraction was carried out using a predefined standardized form, and the collected information was entered into a customized Excel^®^ spreadsheet (Microsoft Corp., Seattle, WA, USA). One reviewer (V.S.) extracted the study data, and a second reviewer (G.S.) checked the accuracy of the extracted information. The following variables were collected: first author, year of publication, country, study design, number of MEC patients, mean age at diagnosis, follow-up duration, tumour size, histologic grade, T and N stage at presentation, surgical treatment, adjuvant therapy, margin status, facial nerve preservation, recurrence, perineural invasion, and lymphovascular invasion.

### 2.6. Data Synthesis and Analysis

All studies included in the qualitative synthesis were also eligible for quantitative analysis. Outcomes were analyzed as reported in the original articles, and when only median follow-up was available, it was used as a proxy for the mean.

A single-arm meta-analysis was conducted to estimate 1-, 5-, and 10-year overall survival (OS) and disease-specific survival (DSS), as well as pooled rates of local, regional, and distant recurrence. Summary estimates were presented with 95% confidence intervals (CIs) and illustrated using forest plots.

As not all included studies reported all outcomes of interest, each meta-analysis was conducted using only studies with available data for the specific endpoint. Consequently, the denominators differed across analyses, and pooled results were presented as meta-analytic estimates with 95% confidence intervals rather than as simple raw proportions.

Between-study heterogeneity was assessed using Cochran’s Q test and quantified with the I^2^ statistic. According to the Cochrane criteria, values from 0% to 40% may represent low heterogeneity, 30% to 60% moderate heterogeneity, 50% to 90% substantial heterogeneity and 75% to 100% considerable heterogeneity.

A random-effects model was applied to account for expected clinical and methodological variability across studies.

To stabilize variance in the presence of extreme proportions, the Freeman–Tukey double arcsine transformation was used. Meta-regression was not performed due to the limited availability of consistently reported covariates across studies.

All the analyses were performed using the R software for statistical computing (R version 4.4.2; “meta” and “dmeta” packages).

### 2.7. Risk of Bias Assessment

The methodological quality of the included studies was independently assessed by two reviewers (V.S. and G.S.) using the Newcastle–Ottawa Scale, as all eligible studies were observational in design. Sensitivity analyses were performed when more than four studies were available for a given outcome, in order to evaluate the robustness of the pooled estimates and explore the influence of individual studies. Publication bias was examined by funnel plot inspection and Egger’s regression test when at least six studies were available for analysis.

## 3. Results

### 3.1. Study Selection

**Figure 1:**
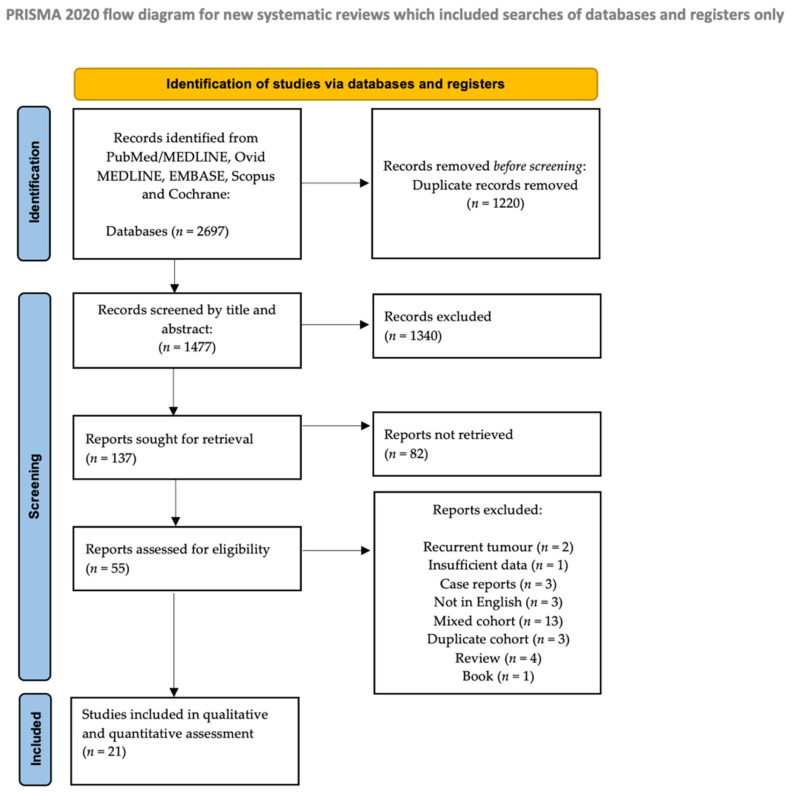
PRISMA flow diagram.

The study selection process is outlined in Figure 1. After removal of duplicate records, 1477 articles remained. Screening by title reduced this number to 137 studies, which then underwent abstract review. Following abstract screening, 55 full-text articles were assessed for eligibility. Of these, 1 article was excluded because it was a book chapter; 3 were case reports; 4 were review articles; 2 involved duplicate cohorts; 13 included mixed populations without extractable parotid MEC data; 3 were not published in English; 1 lacked sufficient data; and 2 included recurrent tumours. Ultimately, 21 studies met all eligibility criteria and were included in the qualitative synthesis and quantitative meta-analysis.

### 3.2. Description of the Studies

The general characteristics of the studies are shown in Table S1. All the studies were in English, and all were retrospective. Five studies were published in the 2020s [7,8,9,10,11], eight in the 2010s [12,13,14,15,16,17,18,19], six in the 2000s [20,21,22,23,24,25], and two in the 1990s [26,27].

### 3.3. Study Results

#### 3.3.1. Quantitative Analysis

A total of 7192 patients with primary MEC of the parotid gland were included in the quantitative analysis. Half of the patients were male (49.7%), with a mean age of 46.6 years (95% CI: 38.8–54.4). At the time of diagnosis, histological grading was low in 1654 patients (32.2%), intermediate in 2150 (41.8%), and high in 1344 (26.1%). The mean tumor size, reported in four studies [11,17,19,20], was 2.31 cm.

Regarding tumor stage, T1 was observed in 1636 patients (46.7%), T2 in 968 (27.6%), T3 in 507 (14.5%), and T4 in 393 (11.2%); nodal stage was N0 in 1137 (60.4%), N1 in 340 (18.1%), N2 in 374 (19.9%), and N3 in 30 patients (1.6%).

Surgical management included total parotidectomy in 2606 patients, superficial parotidectomy in 1642, radical parotidectomy in 9, lobectomy in 5, local excision in 6, subtotal parotidectomy in 1, and no surgery in 66 patients. Facial nerve preservation was achieved in 1993 of 4111 patients, and neck dissection was performed in 1744 patients. Positive surgical margins were reported in 18% of patients. Post operative RT was administered in 3413 patients (50%).

The mean follow-up time was 72.6 months.

**Figure 2:**
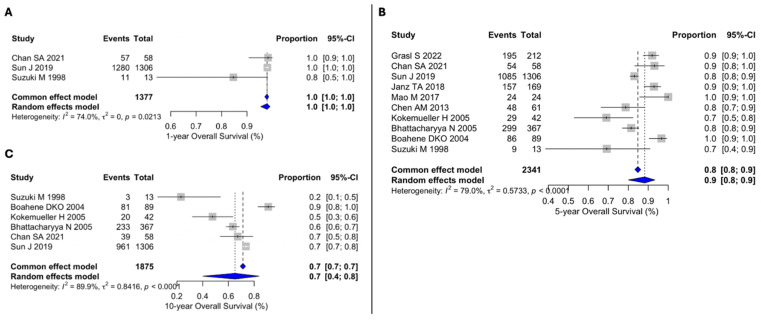
(**A**) Forest plot for 1y-OS. (**B**) Forest plot for 5y-OS. (**C**) Forest plot for 10y-OS. The included studies were Chan et al. [11], Sun et al. [15], Suzuki et al. [26], Grasl et al. [10], Janz et al. [16], Mao et al. [17], Chen et al. [18], Kokemueller et al. [24], Bhattacharyya et al. [20], and Boahene et al. [25]. Grey squares represent individual study estimates, with square size proportional to study weight; grey horizontal lines indicate 95% confidence intervals. Blue diamonds represent pooled estimates, with diamond width indicating the 95% confidence interval. Vertical dashed lines indicate the pooled summary estimate(s). Abbreviations: CI, confidence interval.

Three studies calculated the 1-year OS rate, with a pooled estimate of 100% (95% CI 100–100), and moderate between-study heterogeneity (I^2^ = 74.0%, Q = 0, *p* = 0.0213) (Figure 2A). The 5-year OS rate was 90% (95% CI 80–90), also with moderate heterogeneity (I^2^ = 79.0%, Q = 0.5733, *p* < 0.001) (Figure 2B). The 10-year OS rate was 70% (95% CI 40–80), with high heterogeneity (I^2^ = 89.9%, Q = 0.8416, *p* < 0.001) (Figure 2C).

**Figure 3:**
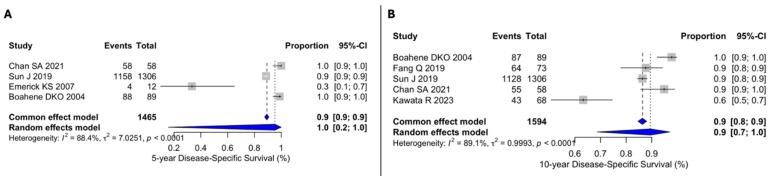
(**A**) Forest plot for 5y-DSS. (**B**) Forest plot for 10y-OS. The included studies were those by Boahene et al. [25], Fang et al. [12], Sun et al. [15], Chan et al. [11], Emerick et al. [21], and Kawata et al. [7]. Grey squares represent individual study estimates, with square size proportional to study weight; grey horizontal lines indicate 95% confidence intervals. Blue diamonds represent pooled estimates, with diamond width indicating the 95% confidence interval. Vertical dashed lines indicate the pooled summary estimate(s). Abbreviations: CI, confidence interval.

Regarding DSS, only Chan et al. [11] reported the 1-year DSS, which was 100%. The pooled 5-year DSS rate of 100% (95% CI 20–100), with high heterogeneity (I^2^ = 88.4%, Q = 7.0251, *p* < 0.001) (Figure 3A). The 10-year DSS estimate across studies was 90% (95% CI 70–100), also with high heterogeneity (I^2^ = 89.1%, Q = 0.9993, *p* < 0.001) (Figure 3B).

**Figure 4:**
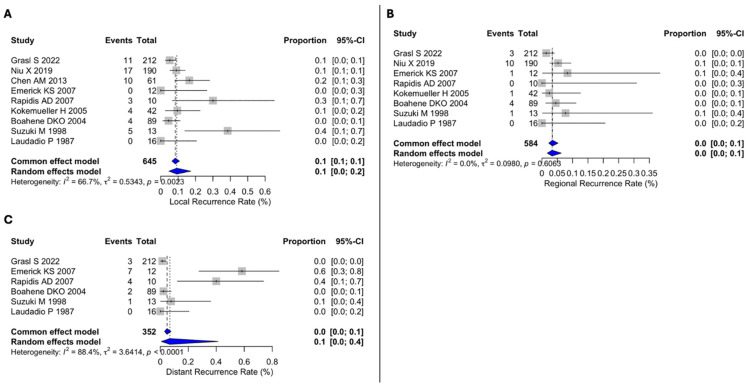
(**A**) Forest plot for local recurrence. (**B**) Forest plot for regional recurrence. (**C**) Forest plot for distant recurrence. The included studies were those by Grasl et al. [10], Niu et al. [13], Chen et al. [18], Emerick et al. [21], Rapidis et al. [22], Kokemueller et al. [24], Boahene et al. [25], Suzuki et al. [26], and Laudadio et al. [27]. Grey squares represent individual study estimates, with square size proportional to study weight; grey horizontal lines indicate 95% confidence intervals. Blue diamonds represent pooled estimates, with diamond width indicating the 95% confidence interval. Vertical dashed lines indicate the pooled summary estimate(s). Abbreviations: CI, confidence interval.

For recurrence, the pooled local recurrence rate was 10% (95% CI 0–20), with moderate heterogeneity (I^2^ = 66.7%, Q = 0.5343, *p* = 0.0023) (Figure 4A). The pooled regional recurrence rate was 0% (95% CI 0–100), based on a limited number of studies. This estimate should be interpreted with caution due to the small number of events and variability in reporting (Figure 4B). The pooled distant recurrence rate was 10% (95% CI 0–40), with high heterogeneity (I^2^ = 88.4%, Q = 3.6414, *p* < 0.001) (Figure 4C).

Grade-stratified survival data were inconsistently reported across studies and were therefore synthesized qualitatively and reported in Table S2.

#### 3.3.2. Qualitative Analysis

Several studies identified prognostic factors associated with survival and recurrence. Age ≥ 65 years, male sex, high histological grade, advanced T or N stage, positive surgical margins, and total parotidectomy were associated with worse OS and DSS in multiple cohorts [8,9,10,11,15]. In particular, intraparotid lymph node (IPN) metastasis and the presence of lymphovascular or perineural invasion were significant predictors of recurrence [13,14]. Several studies reported an association between post-operative RT and improved local control in high-risk patients, particularly in the presence of positive margins, high-grade tumours, or nodal involvement [10,15,19].

##### Outcomes According to Histologic Grade

Histologic grade was reported in most included studies and, across the pooled cohort, high-grade tumours accounted for approximately 26% of cases. Grade-specific survival outcomes were inconsistently reported, limiting formal pooled comparisons. Nevertheless, the available evidence consistently demonstrated inferior outcomes for high-grade MEC. Chen MM et al. [19] reported 5-year DSS rates of 98.8% for low-grade, 97.4% for intermediate-grade, and 67.0% for high-grade tumours. Similarly, Chen AM et al. [18] reported 5-year OS rates of 84%, 80%, and 52% for low-, intermediate-, and high-grade MEC, respectively. In high-grade-only cohorts, survival was also markedly reduced, with Emerick et al. [21] reporting a 5-year DSS of 30%. Overall, these findings support histologic grade as one of the most relevant prognostic determinants in parotid MEC. A summary of grade-specific survival outcomes is provided in Supplementary Table S2.

### 3.4. Risk of Bias Assessment

The Newcastle–Ottawa Quality Assessment Scale scores for the included studies are reported in Table 1.

Leave-one-out sensitivity analyses showed that the pooled estimates for 1-, 5-, and 10-year OS and DSS, as well as for local, regional, and distant recurrence, remained consistent, indicating that the overall results were not driven by any single study (Figure S1).

Funnel plots for each outcome are presented in Figure S2. Visual inspection revealed no meaningful evidence of publication bias or small-study effects across the assessed outcomes.

## 4. Discussion

MEC is the most common malignant histologic subtype of parotid gland tumours, accounting for approximately 50% of all parotid malignancies [28]. The purpose of this systematic review and meta-analysis was to analyse and synthesize the available literature on this rare neoplasm, with the aim of evaluating current treatment strategies, survival and recurrence outcomes, and associated prognostic and risk factors.

The pooled survival estimates of this meta-analysis confirm that MEC of the parotid gland generally carries a favourable short and intermediate term prognosis. The 1-year OS and DSS rates of 100% reflect the typically indolent behaviour of low- and intermediate-grade tumours, which constitute the majority of cases in most cohorts. The 5-year OS of 90% and DSS of 100% indicate that disease-related mortality remains low during the first years after treatment, although overall mortality becomes increasingly influenced by patient-related factors such as age and comorbidities, as highlighted in several included studies [11,15,19,20]. The decline in 10-year OS to 70%, with a more preserved DSS of 90%, further reinforces this pattern: long-term deaths appear to be driven more by competing risks than by MEC itself, except in high-grade or advanced-stage disease. Additionally, it is noteworthy that 26% of patients in this cohort presented with high-grade tumours at diagnosis, highlighting that aggressive histologic subtypes represent a substantial proportion of parotid MEC cases.

The substantial heterogeneity observed across OS and DSS estimates likely reflects differences in tumour grade distribution, treatment strategies, and follow-up length among studies spanning nearly three decades. Cohorts enriched in high-grade tumours or with a higher prevalence of nodal disease reported markedly lower long-term survival, consistent with the known aggressive biology of these subtypes. Variability in the use of adjuvant radiotherapy and elective neck dissection may also contribute to outcome differences. Chen MM et al. [19] identified histologic grade, tumour size, extraparenchymal extension, lymph node metastases, and distant metastases as independent prognostic factors for survival. Bhattacharyya et al. [20] reported age (HR, 1.05; *p* < 0.001), extraparenchymal extension (HR, 3.17; *p* < 0.001), and tumour grade (HR, 1.48; *p* = 0.003) as significant predictors of decreased survival, while Nance et al. [4] found histologic grade and positive margins predicted disease-free survival.

Recurrence analysis revealed a pooled local recurrence rate of 10%, with a similar distant recurrence rate, whereas regional recurrence appeared to be uncommon. These findings underscore the generally localized behaviour of MEC but also highlight that distant metastases, although uncommon, remain a relevant risk, particularly in high-grade tumours or those with nodal involvement. IPN metastasis, lymphovascular invasion, and perineural invasion were significant predictors of recurrence [7,13], suggesting that careful assessment of these features at diagnosis is critical. Fang et al. reported a median time to recurrence of 27 months. Similarly, Chen AM et al. [18] reported a median time to local–regional recurrence of 26 months with all but one of the local–regional recurrences occurring within 5 years of diagnosis.

Surgical management remains the cornerstone of treatment. Total and superficial parotidectomy were the most commonly performed procedures, with facial nerve preservation achieved in a substantial proportion of cases. Positive surgical margins were reported in 18% of patients, reinforcing the challenge of achieving oncologic clearance while preserving function. Post-operative RT was administered in approximately 50% of patients and was associated with improved local control in high-risk populations, including those with positive margins, high-grade tumours, or nodal metastases [10,15].

**Figure 5:**
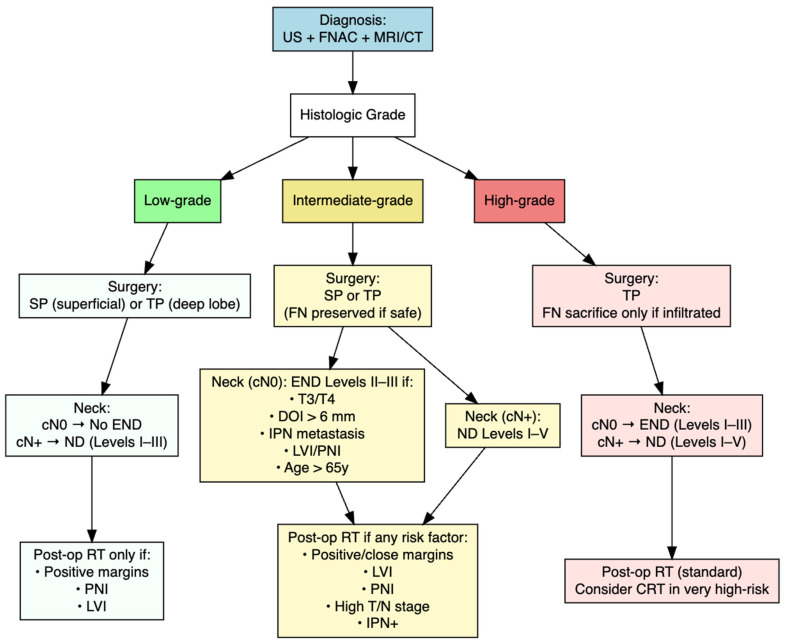
Algorithm for the management of primitive MEC of the parotid gland. Color coding indicates the management pathway according to histologic grade: green, low-grade; yellow, intermediate-grade; red/pink, high-grade; blue, diagnostic work-up. Abbreviations: ECE = extracapsular extension; END = elective neck dissection; FN = facial nerve; LVI = lymphovascular invasion; ND = neck dissection; PNI = perineural invasion; IPN = intraparotid node; RT = radiotherapy; SP = superficial parotidectomy; TP = total parotidectomy.

To date, no standardized or universally accepted guidelines exist to define the optimal management of parotid MEC. In light of the heterogeneity of the available evidence and the absence of prospective data, we present a pragmatic, grade-based treatment framework informed by the literature and institutional clinical experience at the Maxillofacial Surgery Unit, University of Naples Federico II (Figure 5). This proposed algorithm is not derived directly from the present meta-analysis and should not be interpreted as a guideline or prescriptive recommendation, but rather as an illustrative tool to support clinical decision-making.

In this model, low-grade tumours are treated with superficial or total parotidectomy depending on tumour depth. END is avoided in clinically node-negative patients, while clinically positive nodes are managed with therapeutic neck dissection of levels I–III. Postoperative RT is reserved for cases with positive margins, perineural invasion, or lymphovascular invasion. Intermediate-grade tumours follow a similar surgical approach, preserving the facial nerve when feasible. END of levels II–III is recommended in cN0 patients with risk factors such as T3/T4 stage, deep tumour invasion (DOI > 6 mm), IPN metastasis, lymphovascular or perineural invasion, or older age. Clinically positive nodes are treated with therapeutic neck dissection extending to levels I–V. Postoperative RT is indicated in the presence of adverse pathological features. High-grade tumours require total parotidectomy with facial nerve sacrifice only if infiltrated, systematic neck dissection for both cN0 (levels I–III) and cN+ (levels I–V), and postoperative RT, with consideration of chemoradiotherapy in very high-risk cases.

Overall, this framework is intended to provide a structured and reproducible overview of current management strategies, while acknowledging the limitations of the available evidence and the need for prospective validation.

A subset of MECs harbor the specific translocation t(11;19)(q21;p13), leading to the CRTC1/3–MAML2 fusion gene. Although this molecular alteration was originally described mainly in low-grade tumours, later studies demonstrated that it may also occur in a subset of intermediate- and high-grade MECs [29,30,31]. Early reports suggested that MAML2 rearrangement was associated with a more favorable prognosis [32,33]; however, subsequent evidence has been less consistent, and the relationship between translocation status and survival remains controversial [34,35]. Therefore, while molecular profiling may improve tumour classification and biological characterization, the prognostic significance of MAML2 status in parotid MEC is still not definitive. Future studies integrating molecular markers with clinicopathological variables are warranted to refine prognostic models and guide personalized treatment strategies.

This study has several limitations that should be acknowledged. First, all included studies were retrospective, with inherent risks of selection bias, incomplete data, and heterogeneity in reporting. Second, there was considerable variability in tumour grading systems, surgical techniques, and adjuvant treatment protocols across studies, which may have contributed to the observed heterogeneity in pooled survival and recurrence estimates. Third, follow-up durations varied widely among cohorts, potentially underestimating late recurrences or long-term complications. Fourth, the pooled estimate of regional recurrence of 0% should be interpreted cautiously, as it likely reflects the limited number of studies reporting this outcome and the low event rates within those cohorts, rather than the true absence of regional recurrence. This highlights the heterogeneity and incomplete reporting of recurrence patterns across studies.

The use of Freeman–Tukey transformation, although appropriate for extreme proportions, may introduce bias compared to alternative methods. In addition, data on prognostic factors such as intraparotid lymph node metastasis, perineural invasion, and margin status were inconsistently reported, precluding a fully adjusted meta-regression analysis. Therefore, the findings of this meta-analysis should be interpreted as associative rather than causal, given the observational nature of the included studies.

Despite these limitations, the study provides valuable insights into the clinical course, treatment outcomes, and prognostic factors of primary MEC of the parotid gland.

Future research should focus on prospective, multicenter studies with standardized reporting of tumour characteristics, surgical techniques, and adjuvant treatments. Additionally, long-term follow-up studies are needed to capture late recurrences and survival outcomes, which would allow for more robust survival modeling and meta-analytic estimates. Integrating molecular and genetic profiling could further improve prognostication and guide personalized treatment strategies for MEC patients.

## 5. Conclusions

To the best of our knowledge, this is the first systematic review and meta-analysis providing a comprehensive synthesis of treatment strategies, survival and recurrence outcomes, and associated prognostic and risk factors. MEC of the parotid gland generally carries a favourable short- and intermediate-term prognosis, particularly for low- and intermediate-grade tumours. Surgical resection, most commonly total or superficial parotidectomy with facial nerve preservation, remains the cornerstone of treatment. Elective neck dissection may be considered in clinically N0 patients with advanced T stage. Post-operative radiotherapy was associated with improved local control in high-risk cases. High histologic grade, advanced T or N stage, positive surgical margins, and intraparotid lymph node metastasis are key predictors of poorer outcomes and recurrence. Despite long-term survival being generally favourable, careful patient selection and multidisciplinary management are essential, especially for high-grade or advanced-stage disease. Given the retrospective nature of the available evidence and the observed heterogeneity across studies, these findings should be interpreted with caution. Future prospective studies with standardized reporting are warranted to improve prognostic stratification and guide treatment strategies.

## Figures and Tables

**Table 1:** Newcastle–Ottawa Quality Assessment Scale scores of the individual studies. Abbreviations: RSP = retrospective.

No.	Author (Year)	Design	Quality Score	Risk of Bias
1	Kawata R, 2023 [7]	RSP	8	Low
2	Li W, 2023 [8]	RSP	7	Low
3	Al-Qurayshi Z, 2022 [9]	RSP	8	Low
4	Grasl S, 2022 [10]	PSP	6	Some Concerns
5	Chan SA; 2021 [11]	RSP	7	Low
6	Fang Q, 2019 [12]	RSP	7	Low
7	Niu X, 2019 [13]	RSP	8	Low
8	Shang X, 2019 [14]	RSP	8	Low
9	Sun J, 2019 [15]	RSP	7	Some Concerns
10	Janz TA, 2018 [16]	RSP	7	Low
11	Mao M, 2017 [17]	RSP	7	Low
12	Chen MM, 2014 [19]	RSP	7	Some Concerns
13	Chen AM, 2013 [18]	RSP	8	Low
14	Emerick KS, 2007 [21]	RSP	7	Low
15	Rapidis AD, 2007 [22]	RSP	7	Low
16	Rahbar R, 2006 [23]	RSP	8	Low
17	Kokemueller H, 2005 [24]	RSP	7	Some Concerns
18	Bhattacharyya N, 2005 [20]	RSP	7	Low
19	Boahene DKO, 2004 [25]	RSP	7	Low
20	Suzuki M, 1998 [26]	RSP	8	Low
21	Laudadio P, 1987 [27]	RSP	7	Low

## Data Availability

The original contributions presented in this study are included in the article/Supplementary Materials. Further inquiries can be directed to the corresponding author.

## Supplementary Materials

The following supporting information can be downloaded at https://www.mdpi.com/article/10.3390/cancers18071146/s1, Table S1: Summary of included studies; Table S2: Grade-specific outcomes; Figure S1: Leave-one-out sensitivity analysis for studies assessing A—5y-OS; B—10y-OS; C—10y-DSS; D—Local recurrence rate; E—Regional recurrence rate; F—Distant recurrence rate; Figure S2: Funnel plot for studies assessing A—5y-OS; B—10y-OS; C—Local recurrence rate; D—Regional recurrence rate; E—Distant recurrence rate.

## Abbreviations

The following abbreviations are used in this manuscript:

CT	chemotherapy
DFS	disease-free survival
DSS	disease specific survival
H	high
I	intermediate
IPN	intraparotid node
L	low
LN	lymph nodes
LVI	lymphovascular invasion
ND	neck dissection
PNI	perineural invasion
OS	overall survival
RFS	recurrence free survival
RND	radical neck dissection
RSP	retrospective
SP	superficial parotidectomy
SubP	subtotal parotidectomy
TP	total parotidectomy
