# Supplementary material for: Prognostic Factors and Survival Outcomes in Parotid Gland Mucoepidermoid Carcinoma: A Systematic Review with Meta-Analysis and Workflow Proposal
Source: Cancers (Basel). 2026 Apr 2;18(7):1146. doi: 10.3390/cancers18071146 (PMC13072125; doi:10.3390/cancers18071146)
Supplement: Supplementary file 1 [file cancers-18-01146-s001.zip › cancers-4207794-supplementary.pdf]

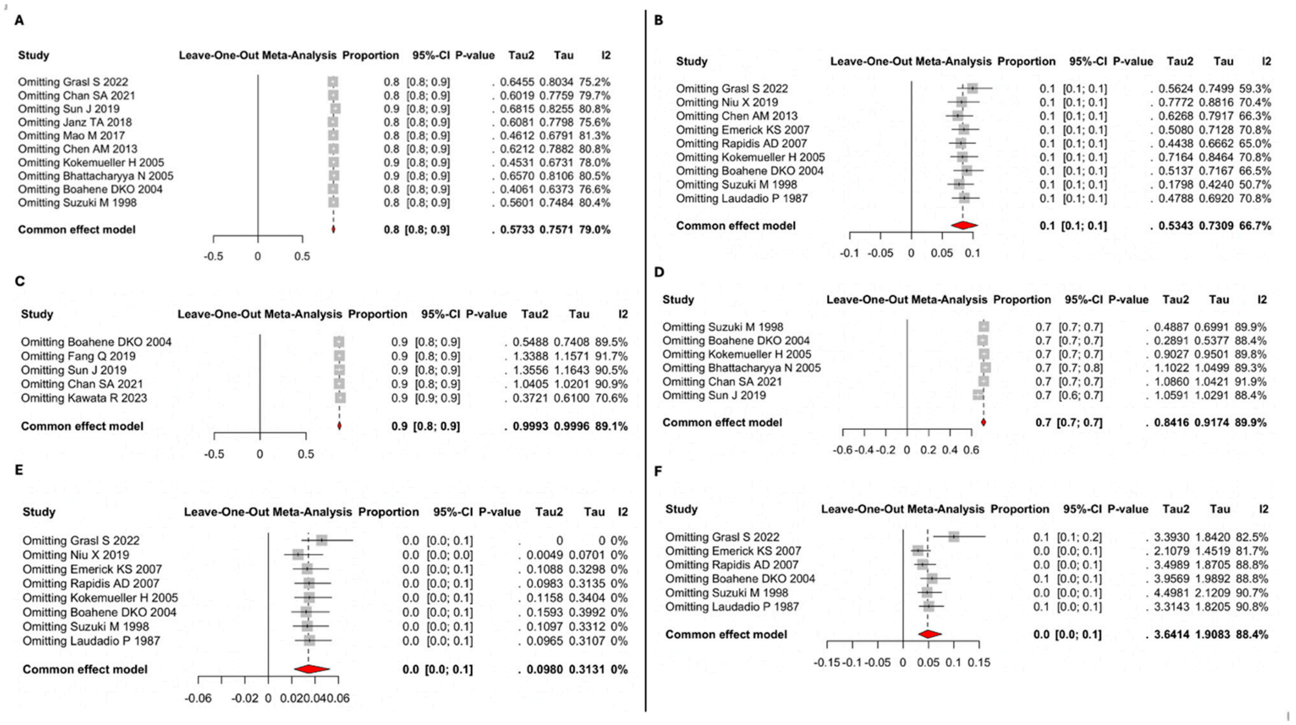

Figure S1 Leave-one-out sensitivity analysis for studies assessing **A-** 5y-OS. B- 10y-OS. **C-** 10y-DSS. **D-** Local recurrence rate. **E-** Regional recurrence rate. **F-** Distant recurrence rate.
Abbreviations: CI = confidential interval

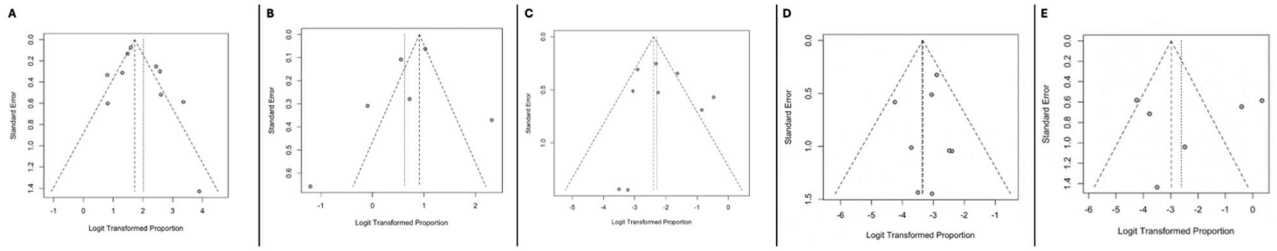

Figure S2 Funnel plot for studies assessing **A-** 5y-OS. B- 10y-OS. **C-** Local recurrence rate. **D-** Regional recurrence rate. **E-** Distant recurrence rate.
Abbreviations: CI = confidential interval

Supplementary Table S1. Summary of included studies.
Abbreviations: * = Median; CT = chemotherapy; DFS = disease free survival; DSS = disease specific survival; H = high; I = intermediate; IPN = intraparotid node; L = low; LN = lymphnodes; LVI = lymphovascular invasion; ND = neck dissection; PNI = perineural invasion; OS = overall survival; RFS = recurrence free survival; RND = radical neck dissection; RSP = retrospective; SP = superficial parotidectomy; SubP = subtotal parotidectomy; TP = total parotidectomy

Author, year	Country	Study Design	Time	MEC (Male)	Mean age at diagnosis (range)	Mean follow up time	Histology Grade	T status	N status	Mean Tumor Size (cm)	Surgery	Margin status	Post-operative treatments	Recurrence	Survival Measures	Outcomes
**Kawata R, 2023 [7]**	Japan	RSP	From September 1995 to December 2022	68	62.7	N/A	H: 31	N/A	N/A	N/A	TP = 24; lobectomy = 5	N/A	N/A	N/A	10y DSS: 63.7%<br><br>10y DFS: 34.9%	Patients with recurrence was significantly higher among those with pain/tenderness and lymph node metastases (p = 0.032 and p = 0.035, respectively)
**Li W, 2023 [8]**	China	RSP	From January 1995 to January 2022	720 (316)	56 ± 16	N/A	L: 172; I: 264; H: 284	T1: 106; T2: 151; T3: 247; T4: 216	N1: 210; N2a: 35; N2b: 209; N3a: 13; N3b: 12	N/A	TP<br><br>ND I-III/IV: 487<br>ND I-V: 233	Positive: 28<br>Negative: 692	RT: 720; 103: CT	N/A	N/A	TNM stage, pathologic grade, IPN metastasis, positive margin, lymph node size, and the ratio of positive to total LNs were significantly associated with OS (p<0.001)
**Al-Qurayshi Z, 2022 [9]**	USA	RSP	From 2004 to 2016	1233 (479)	N/A	57.69mo* (35.71–91.30)	L: 581; 493: I; 159: H	T1: 784; T2: 354; T3: 68; T4a: 27	N0	N/A	SP: 717; TP: 516<br><br>ND: 970	Positive: 235<br>Negative: 998	RT: 440	N/A	N/A	Among patients with no adverse pathological features, age 65 years and older (adjusted HR 4.58, 95%CI 2.77–7.59; p < 0.001), male sex (adjusted HR 2.22, 95%CI 1.36–3.60; p = 0.001), and total parotidectomy (adjusted HR 4.06, 95%CI 1.61–10.26; p = 0.003) were associated with worse OS. With respect to patients with one or more advanced

																pathological features, age 65 years and older (adjusted HR 6.13, 95%CI 3.59–10.45; p < 0.001), Charlson/Deyo score ≥2 (adjusted HR 2.96, 95%CI 1.24–7.08; p = 0.014), and total parotidectomy (adjusted HR 2.26, 95%CI 1.07–4.78; p = 0.34) were associated with worse OS. Comparing the two groups, OS was worse among patients with one or more advanced pathological feature.
**Grasl S, 2022 [10]**	Multicentric	RSP	From 1990 and 2020	212 (102)	47	45mo*	L 105; I: 73; H: 34	T1: 97; T2: 68; T3: 26; T4: 21	N0: 58; N+: 40	N/A	TP	Negative: 134 Positive: 66 N/A.: 12	RT: 127	11: local; 3: regional; 3: distant; 1: regional/distant; 2: locoregional<br><br>5-year local, regional, and distant control rates were 92.1%, 96.4%, and 97.5%, respectively	5y OS: 91.8% 5y DSS: 95.2% 5y DFS: 87.9%.	3 tumor and presence of LVI or ECE were the only factors that were significantly associated with worse OS (p < 0.001 each), DSS (p < 0.001 each), and DFS (p < 0.001 each) (Fig. 2). T3/4 compared to T1/2 staged carcinomas had a significantly worse 5-year OS (77.9% vs. 95.9%, p = <0.001) as well as DSS (81.9% vs. 99.1%, p = <0.001). Presence of lymph node metastases was

																significantly associated with a lower OS (70.7% vs. 100%, p = <0.001) and DSS (82.8% vs. 100%, p = <0.001) rate compared to patients that were pN0.
**Chan SA; 2021 [11]**	USA	RSP	From 1995 and 2014	58 (19)	56* (16-87)	5.9y (0-20)	L 27; I: 17; H: 12	T1: 35; T2: 14; T4a: 9	N0: 53; N1: 2; N2b: 3	1.9cm (TP) vs 1.5cm (SP)	TP: 27; SP: 31<br><br>ND: 19	N/A	RT: 8	loco-regional: 2<br><br>1y RFS: 98%<br>5y RFS: 95.3%<br>10y RFS: 95.3%<br>15y RFS: 95.3%	1y OS: 98.1%<br>5y OS: 93.6%<br>10y OS: 67.4%<br>15y OS: 47.1%<br><br>1y CSS: 100%<br>5y CSS: 100%<br>10y CSS: 94.1%<br>15y CSS: 94.1%	significant association of age with survival (HR 1.08, p = 0.009)
**Fang Q, 2019 [12]**	China	RSP	From January 1990 to December 2017	73 (30)	14.3 (8-18)	95.4mo (13-286)	L 42; I: 20; H: 11	T1: 40; T2: 19; T3: 10; T4: 4		N/A	TP	Positive: 5<br>Negative: 68	RT: 15<br>CT+RT: 4	loco-regional: 12<br><br>10-year LRC rate was 83%, and the median time to recurrence was 27 (13–80) months.	10y DSS: 88%	IPN metastasis remained significantly related to the disease-related death
**Niu X, 2019 [13]**	China	RSP	From January 2001 to December 2017	190 (89)	48.7 (18-85)	71.1mo	L 104; I: 62; H: 24	T1/2: 122; T3/4: 68	N0: 6; N+: 30	N/A	N/A	N/A	RT: 65	Local: 17; neck: 10; loco-regional: 7<br><br>10-year RFS: 79%<br><br>10-year RFS rate was 54% for patients with IPN metastasis, and 88% for patients without IPN metastasis (p < 0.001)	N/A	Tumor stage, IPN metastasis, PNI, and LVI as well as disease grade were significantly associated with the recurrence (p < 0.05)
**Shang X, 2019 [14]**	China	RSP	From January 2000 to December 2016	122 (55)	58.5 (19-83)	69.7mo (5-201)	H	T1/2: 45; T3/4: 77	N0: 50; N+: 72	N/A	TP	Positive: 9<br>Negative: 113	RT: 122; RT+CT: 35; RT+CT+Trastuzumab: 8	In patients without IPN metastasis, the 5-year RFS rate was 83%; in patients with only superficial	N/A	Tumor stage and cervical lymph node stage were statistically related to lymph node metastasis in the deep lobe,

														lobe parotid metastasis, the 5year RFS rate was 56%; in patients with only deep lobe parotid metastasis, the 5-year RFS rate was 24%; in patients with superficial and deep lymph node parotid metastasis, the 5-year RFS rate was 11% (P < 0.001)		and a similar relation was found for the development of lymph node metastasis in the superficial lobe.
**Sun J, 2019 [15]**	China	RSP	From 2004 to 2015	1306 (646)	53 (19.6)	54mo* (4-143)	Different grading system	T1: 605; T2: 363; T3: 188; T4a: 129; T4b: 21	N0: 1058; N1: 124; N2a: 6; N2b: 110; N2c: 3; N3: 5	N/A	1286	N/A	RT: 609<br>CT: 81	N/A	5y OS: 83.1%<br>10y OS: 73.6%<br><br>5y CSS: 88.7%<br>10y CSS: 86.4%<br><br><u>T1 stage</u><br>5y OS: 93.3%<br>10y OS: 87.3%<br><br><u>T2 stage</u><br>5y OS: 86.2%<br>10y OS: 75.1%<br><br><u>T3 stage</u><br>5y OS: 65.8%<br>10y OS: 53.0%<br><br><u>T4 stage</u><br>5y OS: 58.7%<br>10y OS: 45.4%<br><br><u>N0 stage</u><br>5y OS: 90.0%<br>10y OS: 81.3%<br><br><u>N1 stage</u><br>5y OS: 59.6%<br>10y OS: 48.0%<br><br><u>N2 stage</u><br>5y OS: 49.2%<br>10y OS: 34.6%<br><br><u>M0 stage</u>	Age (p < 0.001), sex (p < 0.001), race (p = 0.006), grade (p < 0.001), TNM stage (p < 0.001), T stage (p < 0.001), N stage (p < 0.001), M stage (p < 0.001), RT (p < 0.001), CT (p < 0.001), and surgery (p < 0.001) were significant prognostic factors of OS<br><br>Age (p < 0.001), sex (p < 0.001), race (p = 0.010), grade (p < 0.001), TNM stage (p < 0.001), T stage (p < 0.001), N stage (p < 0.001), M stage (p < 0.001), radiotherapy (p < 0.001), chemotherapy (p < 0.001), and surgery (p < 0.001) were significant

															5y OS: 84.3%<br>10y OS: 74.6%	prognostic factors for DSS
**Janz TA, 2018 [16]**	USA	RSP	From 1973 to 2014	169 (78)	13.4 (3.0–18.0)	N/A	N/A	Tx: 6; T1: 25; T2: 34; T3: 14; T4: 7	N/A	N/A	161	N/A	RT: 54	N/A	2y OS: 98%<br>5y OS: 93%<br><br>2y DSS: 99%<br>5y DSS: 98%	Neither 5y OS or DSS significantly changed over time, with all cohorts having greater than 90% 5-year OS and DSS rates
**Mao M, 2017 [17]**	China	RSP	From September 2002 to January 2012	24 (12)	13.2 (5-16)	7.2y* (5–13.4)	L 7; I: 15; H: 2	T1: 2; T2: 17; T3: 1	N/A	2.4cm	Conservative surgery with preservation of the facial nerve	Positive: 12<br>Negative: 12	^{125}I seed brachytherapy	4	5y OS: 100%.<br>5y DFS: 100%.	N/A.
**Chen MM, 2014 [19]**	USA	RSP	From 1998 to 2009	2400 (1408)	52.8(L) vs 52(I) vs 66(H)	N/A	L: 522; I: 1137; H: 741	N/A	N/A	2.0cm(L) vs 2.1(I) vs 3.2(H)	SP: 809;<br>TP: 812; no surgery: 65	N/A	RT: 1112	N/A	<u>5y DSS</u><br>L: 98.8%<br>I: 97.4%<br>H: 67.0%<br><br>but high-grade MEC exhibited reduced survival when compared with low/intermediate-grade MEC (67.0% vs 97.8%; p < .001	5y DSS between low grade and intermediate-grade MEC was similar (p = 0.09), but high-grade MEC exhibited reduced survival when compared with low/intermediate-grade MEC (p < 0.001)<br><br>Univariate analysis, patient sex, age, race, grade, tumor size, extraparenchymal extension, distant metastases, positive lymph nodes, type of surgery, and EBRT were associated with decreased survival<br><br>Multivariate analysis, high-grade histology (HR, 5.66; p < 0.001), increasing patient age (HR, 1.02; p < 0.001), increasing tumor

																size (2–4 cm; HR, 1.58; p = 0.04; 4 cm; HR, 1.79; p = 0.03), extraparenchymal extension (HR, 1.88; p = 0.001), positive lymph nodes (HR, 2.86; p < 0.001), and distant metastases (HR, 5.80; p = 0.004) were independently associated with decreased DSS in MEC
**Chen AM, 2013 [18]**	USA	RSP	From 1998 to 2008	61 (36)	60* (30-94)	45mo* (6-101)	L 18; I: 19; H: 24	T1: 14; T2: 14; T3: 14; T4: 19		N/A	SP: 35; TP: 20; local excision: 6<br><br>ND: 3	Positive: 9; Negative: 52	RT: 61	3y and 5y estimates of local-regional control were 89% and 84%, respectively<br><br>Median time to local-regional recurrence: 26mo (6-75) with all but one of the local-regional recurrences occurring within 5y from diagnosis<br><br>5y rates of local regional control for patients with T1, T2, T3, and T4 tumors were 92%, 83%, 83%, and 64%, respectively (p = 0.09)<br><br>Distant: 20	3y OS: 85%<br>5y OS: 79%<br><br><u>5y OS</u><br>L: 84%<br>I: 80%<br>H: 52%	high tumor grade, T4 disease, and pathological lymph-node metastasis predicted for decreased survival<br><br>On univariate analysis, T4 disease was predictive of local-regional recurrence<br><br>5y OS for patients with low, intermediate, and high-grade tumors (p = 0.08)
**Emerick KS, 2007**	USA	RSP	From 1977 to 1997	12 (10)	62	(41-103)mo	H	T1: 1; T2: 2; T3: 2; T4: 7	N0: 7; N2b: 5	N/A	TP: 6; SubTP: 1; SP: 4; no surgery: 1<br><br>modified RND: 4	N/A	RT: 11	Neck: 1; Distant: 7<br><br>Overall regional control	Survival time in months ranged from 5 to 35 months with a mean of 20	in terms of survival, the odds of dying when neck metastases occurred was 5.3

														was achieved in 9/11 patients	5y DSS: 30%<br>5y DFS: 20%	times greater than in N0 patients
**Rapidis AD, 2007**	Greece	RSP	From January 1994 to December 2004	10	59.3 (29-86)	(6-120)mo	L: 5; I: 2; H: 3	N/A	N/A	N/A	SP: 3; TP. 6; hemimandibulectomy: 1<br><br>supraomohyoid ND: 2; RND: 2	Positive: 3 Negative: 7	RT: 5	Local: 3; Distant: 4	DFS 4 su 5<br><br>1 AWD<br><br>2: DOC<br>1: DOD<br><br>Mean DFS of 36mo<br><br>Mean OSS of 53mo	The main parameter significantly affecting survival was the histological grade of tumors (p = 0.013)
**Rahbar R, 2006**	USA	RSP	From 1994 to 2004	7 (3)	11.4 (3-15)	3.4y	L	T1: 1; T2: 3; N/A: 3		N/A	TP: 3; SP: 3<br><br>supraomohyoid ND: 1	Positive: 4 Negative: 3	RT: 1	1	N/A	N/A
**Kokemueller H, 2005**	Germany	RSP	From January 1983 to December 2002	42	50*	8.9y<br>L: 10.5y vs H: 3.1y	L: 6 H: 5	T1: 4; T2: 2; T3: 3; T4: 3	N0: 8; N1: 2; N2: 1	N/A	Incomplete resection: 5	Positive: 2 Negative: 9	RT: 3	Local: 4<br>Regional: 1	5y OS: 68.8%<br>10y OSS: 48.2%<br><br>Mean survival time of 12.7y	Grade (p < 0.001), stage (p < 0.001) and margin status (p < 0.001) significantly influenced prognosis.
**Bhattacharyya N, 2005 [20]**	USA	RSP	From 1988 to 1998	367	59.2	51.8mo	N/A	N/A	N/A	2.7 cm	N/A	N/A	N/A	N/A	5y OS: 81.5%<br>10y OS: 63.5%<br><br>Mean survival time of 105mo	tumor grade remained a statistically significant predictor of survival<br><br>age, tumor size, tumor grade, extraglandular extension, positive cervical nodes, and use of external-beam radiation therapy statistically influenced survival
**Boahene DKO, 2004 [25]**	USA	RSP	From 1940 to 1994	89 (33)	49 (0-36.9)	14.7y	L 43; I: 40; H: 6	N/A	N/A	N/A	RP: 9; TP: 48; SP: 32<br><br>ND: 15	N/A	RT: 7	Local: 4<br>Regional: 4<br>Distant: 2	5y OS: 96.6%<br>10y OS: 91.3%<br>15y OS: 79.5%,<br>20y: 63.8%<br>25y OS: 54.1%<br><br>5y DSS: 98.8%<br>10y DSS: 97.4%<br>15y DSS: 97.4%	N/A

															20y DSS: 97.4%<br>25y DSS: 97.4%,	
**Suzuki M, 1998 [26]**	Japan	RSP	From 1981 to 1997	13 (11)	56.4 (40-78)	61.8mo (9-174)	L: 4; I: 4; H: 5	T2a: 6; T2b: 1; T3a: 1; T3b: 3; T4a: 2	N0: 9; N1: 2; N2b: 2	N/A	TP: 9; PT: 4<br><br>ND: 9	N/A	RT: 6<br>CT: 6	Local: 5; Neck: 1; Lung: 1	1y OS: 85%<br>5y OS: 69%<br>10y OS: 26%<br><br><u>1y OS</u><br>Stage I: 100%<br>Stage II-III: 100%<br>Stage IV: 0%<br>L: 100%<br>I: 100%<br>H: 60%<br>HER-2/neu (-)/(+): 100%<br>HER-2/neu (++): 50%<br><br><u>5y OS</u><br>Stage I: 100%<br>Stage II-III: 67%<br>Stage IV: 0%<br>L: 100%<br>I: 100%<br>H: 0%<br>HER-2/neu (-)/(+): 89%<br>HER-2/neu (++): 25%<br><br><u>10y OS</u><br>Stage I: 100%<br>Stage II-III: 0%<br>Stage IV: 0%<br>L: 0%<br>I: 50%<br>H: 0%<br>HER-2/neu (-)/(+): 33%<br>HER-2/neu (++): 0%	patients with high malignancy tumours (grade 3 tumours with strong HER-2/neu) had shorter recurrence-free intervals and shorter OS than the patients with low malignancy tumours (tumours of grade 1 or 2 and low (no, trace positive, or moderate) HER-2/neu expression)
**Laudadio P, 1987 [27]**	Italy	RSP	From 1968 to 1983	16 (6)	45 (11-73)	Minimum 5y	L: 9; I: 4; H: 3	Tx: 2; T1: 6; T2: 4; T3: 3; T4: 1	N0	N/A	TP: 8; SP: 8	N/A	None	0	9 alive with no disease<br>2 deaths due to distant metastasis	N//A

Supplementary Table S2. Grade-specific outcomes

Study	*Outcome*	*Low-grade*	*Intermediate-grade*	*High-grade*
Chen MM 2014	5y DSS	98.8%	97.4%	67.0%
Chen AM 2013	5y OS	84%	80%	52%
Suzuki 1998	5y OS	100%	100%	0%
Emerick 2007	5y DSS	–	–	30%
Kawata 2023	10y DSS	–	–	63.7%
